# Is Assisted Dying Really a Matter for Medical Regulation?

**DOI:** 10.1017/jme.2025.10117

**Published:** 2025

**Authors:** Jennifer Hardes Dvorak

**Affiliations:** Law, Canterbury Christ Church University, Canterbury, United Kingdom

**Keywords:** assisted dying, demedicalization, medicalization, pharmaceuticalization, regulation

## Abstract

This paper considers whether assisted suicide and euthanasia (AS/E) is an area for medical regulation or whether there is a better alternative regulatory mechanism to govern it. Drawing from empirical evidence across a range of jurisdictions where it is legalized, the paper argues that there are at least four good reasons to consider demedicalizing AS/E: (1) pragmatic ethical issues of infrastructural weakness in AS/E service provision in already overstretched healthcare systems globally; (2) challenges of medicalization; (3) regulatory complexities concerning medical law (including pharmaceutical law) and criminal law; (4) the risk that AS/E becomes more easily susceptible to healthcare economics. The paper suggests several recommendations concerning a possible “demedicalized model.”

## Introduction

In his recent *Bioethics* editorial, Udo Schuklenk^1^ asks whether it is time to rethink assisted dying (AD). Arguing that we have moved past the deeper moral question of whether we *ought* to permit assisted dying, Schuklenk suggests the question for scholars to respond to is now a matter of “how” rather than “if.” I agree that regulation is key: there is little chance of reversing the tide of assisted dying legalization, which has spread rapidly across the global north. Effort is better spent on the pragmatic questions concerning how best to ensure a safe and effective process of assisted dying. Schuklenk suggests that there are at least three questions that ought to concern those interested in regulation: eligibility criteria, advance directives and the realm and remit of healthcare professionals. I leave advance directives for another day, but in this paper focus on questions one and three. These questions are, arguably, intimately linked, bound within a broader point I raise here in relation to medicalization. This paper focuses on the question of whether assisted dying really is a medical issue or whether it might be better regulated outside of healthcare, a particularly pertinent question because in most jurisdictions where assisted dying has been legalized (either in the form of assisted suicide or euthanasia, herein referred to as AS/E), the task falls onto doctors, and medicine provides “the main frame of reference.”^2^ Some states have extended eligibility to perform AS/E to nurse practitioners (for example, Canada, Australia, and many US states). In some exceptional cases, namely in Switzerland, those volunteering for “right to die” organizations perform assisted suicide (AS, not E) and doctors are distanced from provision. Yet, as this paper later argues, doctors remain intimately bound within assisted suicide processes, chiefly because of the complexities of pharmaceutical laws. To this end, I argue that any “demedicalized” form of AS/E provision would need to account not only for the challenges posed by medicalization, but also those of pharmaceuticalization.

To frame the paper, I outline four inter-related challenges that medical models of AS/E pose. First, I consider the pragmatic issue of possible doctor objection, and the issue of efficient AS/E provision. Next, I consider the “medicalization” of assisted dying which encompasses: (a) the notion of eligibility criteria, and the expansion of medical criteria to govern otherwise “normal” areas of life (also known as the “bracket creep”) and (b) the prospect that medicalization depoliticizes social inequalities in healthcare which ought to instead be emphasized as “public issues.” Third, I examine the regulatory complexities of the continued role of criminal law, the role of overlapping areas of medical law (including pharmaceutical laws), and the prospect of criminal prosecution for doctors. Finally, I examine the problem of healthcare economics.

In examining these four points I draw primarily on two jurisdictions as guiding case studies — the Netherlands, which has emerged as a well-established medical model of AS/E, and Switzerland, the only country worldwide to establish a “demedicalized” model of assisted suicide. For reference, I also draw from data and cases from other jurisdictions such as Canada and Australia, which have implemented broader healthcare models to encompass the assistance of nurse practitioners as well as doctors in their AS/E provision. The paper asks whether assisted dying can and should be “demedicalized” and concludes with some tentative regulatory recommendations.

## Pragmatic issues with medical models

The involvement of healthcare professionals in assisted dying service provision is not without criticism, nor is it without objection.^3^ In Britain, where I am currently writing from, survey data reveals that although the British Medical Association (BMA) has shifted its stance on assisted dying from “opposed” to “neutral,” a stance that opens the door for legal change here, specialisms most opposed to involvement in assisted dying are general practitioners, geriatric specialists and palliative care doctors, namely those specialisms who are most likely to be tasked with performing assisted dying in any legalized pathway.^4^ European data from palliative care specialists reveals similar opposition from this group of practitioners, who are arguably the most experienced in caring for patients at the end of life.^5^

One cannot ignore possible professional resistance when considering the best regulatory model for AS/E. Because medical models of assisted dying require doctors to perform it, conscientious objection is a significant barrier to regulatory efficiency.^6^ Research evidence reveals that almost half of doctors struggle with performing AS/E and are uncomfortable about their participation in it.^7^ Qualitative research with Canadian physicians reveals feelings of emotional burden and psychological impact,^8^ corroborated in further studies.^9^ This emotional burden is shared by Dutch general practitioners, who report similar feelings, in a jurisdiction where AS/E has been practiced for a much longer period of time and is therefore an even more normalized part of healthcare.^10^

Conscientious objection (CO) clauses are built into all AS/E regulatory regimes to protect against doctors’ personal moral stance on the topic. Although important, CO clauses do not protect against two discrete issues: first, if too many doctors object there may be inadequate provision of the service. Second, CO clauses do not necessarily protect against other institutional pressures such as coercion in systems that are already overburdened, and which may pressure doctors to perform AS/E. Laws that lack the infrastructure to bring them into being also risk other possible consequences. Significant research already exists on the pragmatic problems of CO in healthcare.^11^ Research from the Netherlands and from Belgium, where AS/E has long been established, reveals that physician objection may lead to the procrastination of cases.^12^ Queensland,^13^ Victoria,^14^ and Western Australia’s^15^ annual assisted dying (AD) review boards report further challenges of AD provision for doctors who do so as part of their current healthcare roles and who are even more stretched as a result. Australian doctors reveal challenges of provision when some choose not to provide this service and it is seen to undermine the doctor-patient relationship.^16^ A more fundamental legal issue is also whether CO clauses can be challenged. The United Nations International Covenant on Civil and Political Rights affirms in Article 18(1)1, for example, the right to CO, yet this is limited by Article 18(3) of this same covenant if it infringes on the fundamental rights of others.^17^ In different jurisdictions, Article 18(3) is given different weight. For example, as Schuklenk notes, the EU Court of Human Rights as well as different domestic courts have tested the right to conscientiously object, with some states being more unwavering in support of this right (e.g., US states) while others, like Sweden, do not permit CO at all. Some critics have suggested that CO and healthcare are “incompatible” because the former does not allow medical professionals to fulfill their professional (and legal) obligations.^18^

Critics of CO also argue that doctors joining a profession should place the public good and rule of law above their own sectarian interests.^19^ The stance that doctors who do not like the law should simply not join the profession is a contentious one. So is the prospect of removing CO that would force doctors who have been practicing but who object to AS/E to be compelled to make an early exit from their careers. In reality, it is only in Sweden that doctors are not entitled to draw on CO on account of professional responsibility and its alignment with the authority of the rule of law; almost all other legalized jurisdictions worldwide permit CO.^20^ More philosophically, though, one can argue that the matter of contention is not restricted to the practical reality of who enters the profession but also to what amounts to the “public good” in the first instance, and how this is determined in the AS/E debate.

Professional responsibility arguments also simplify the conception of how power and authority operate. It is arguably impossible to protect against workplace hierarchies and power relations where doctors may be bullied in professional settings for refusing to participate in AS/E, an issue that affects junior doctors in particular.^21^ In Canada, doctors objecting to take part in AS/E have reported bullying and harassment in their workforce, and many have reported being forced out of their jobs to seek employment in other areas because of fear of persecution for their moral objections to AS/E.^22^ Even if some doctors are in support of AS/E provision, a critical mass of staff is needed for service delivery. Staffing challenges have led to Australian AS/E reviews noting the need for better remuneration for staff who end up taking on AS/E provision on top of their normal job roles.^23^ In Oregon, only 2% of doctors in 2022 prescribed lethal drugs and the prescriber was only present in 13% of deaths.^24^

Concerns over the practice of “doctor-shopping” where patients have to locate willing providers is accompanied by “multiple prescribing”: Preston notes that in Oregon, the 2016 report records that “one doctor wrote 25 prescriptions for lethal drugs in that year.”^25^ A recent Canadian example of doctor shopping saw a British Columbia judge issue an interim injunction to block a doctor from providing Medical Assistance in Dying (MAiD) to a 53-year-old woman. The patient claimed to suffer with akathisia, characterized by restlessness, terror, agitation, an inability to sit still, and burning skin sensations, symptoms which the judge noted were connected to the changes in the usage of drugs connected to her underlying psychological condition of bipolar disorder. Doctors in the woman’s home province of Alberta had deemed her ineligible for MAiD as she did not meet the legislative requirements of an “irremediable” condition and there were concerns over her “unresolved mental health problems.” However, she had been able to seek out a well-known doctor and MAiD provider in the neighboring province of British Columbia who had agreed to euthanize her.^26^

Conversely, one might argue that if AS/E is sequestered from medical care, those doctors who do choose to opt in may also feel marginalized by colleagues.^27^ Some British palliative care doctors, for example, have reported feeling ostracized by the palliative care community at large which tends to remain one of the most opposed specialisms globally against AS/E.^28^ Since data from the British palliative care community shows that only 4% of this specialism are in support of AS/E,^29^ it is possible that the minority in favor would feel silenced and compelled not to outwardly support AS/E even if it were legalized for fear of judgment from colleagues, which may in turn also impact professional opportunities to progress in one’s field.

Given the ethical and practical challenges to delivery, it may be more pragmatic to consider separating AS/E provision from healthcare. An opt in model could be offered outside of normal healthcare and as a separate service that is properly given time and remuneration for those who want to participate in it. Opening this role to non-medics would also give relief from doctors and opportunities to AS/E advocates who would feel less emotionally burdened by their participation in service delivery. It would also enable those doctors who support AS/E to do so openly without fear of judgment, particularly those who want to work in palliative care, an area that tends to be most opposed to AS/E. It would potentially make the service more efficient because professionals could be trained to perform it. Switzerland stands as an example to show that medics are not required to perform AS/E.

## AS/E “medicalization”

Besides the pragmatic issues of objection and provision when framed inside of healthcare, there are theoretical critiques of medical involvement in AS/E regulation. From a sociological perspective, “medicalization” refers to the process of transforming social problems into medical problems. In the case of AS/E, medicalization refers to the shifting management of dying — and thereby assisted dying — into the domain and management of medicine. Indeed, as Szasz commented, “Suicide began as a sin, became a crime, then became a mental illness, and now some people propose transferring it into the category called “treatment,” provided the “cure” is under the control of doctors.”^30^ The westernized account of a “good death” being pain free, aware (decided and under control) and that meets one’s personal preferences and individual choice is decidedly modern. As Clark^31^ argues, it is the process of dying rather than death itself which is medicalized and which people seek refuge from. Where palliative care is one option for managing death, AS/E risks being seen to offer a magic bullet — a quick and efficient means to end suffering under one’s control.^32^

Historically, medicalization has often been construed as a “dirty word,” implying that medicine has been used as a means of inappropriately exercising social control^33^ or as a form of medical imperialism.^34^ However, the concern for AS/E is arguably not the prospect of the power of doctors and their authority but more so the matter that a significantly large percentage of doctors often do not want AS/E within their jurisdiction, as the first section of this paper highlights.

Indeed, some sociologists have acknowledged medicalization’s advantages.^35^ Bringing certain areas under medical jurisdiction can destigmatize individuals and create a safe space. For instance, reframing suicide or labelling people with “medical conditions” like attention deficit hyperactivity disorder (ADHD) can remove markers of deviancy^36^ and can normalize behaviors or traits that might otherwise be seen as marginalized and thus stigmatized.^37^ Medicalizing AS/E might foster the sentiment that death need not be treated as a taboo, as something that must be resisted, postponed or avoided, but instead may be treated as a topic to be openly discussed, managed, regulated, and planned for. Medicalization may also provide a protective barrier against a “slippery slope” that may otherwise see expanding criteria for AS/E beyond those who are terminally ill or already very sick to “anyone.” Because doctors limit their care to the sick, this should, in theory at least, protect against a “bracket creep.”^38^ Thus, medicalization has the advantage of restricting eligibility criteria.

However, in practice the regulation of AS/E under medical control arguably complicates rather than clarifies eligibility criteria. Medical involvement in AS/E regulation presents two problems concerning eligibility: (1) medical models tend (initially) to restrict eligibility to medical illness and thereby sometimes they are accused of discriminating against persons who seek AS/E but do not fit strict medical eligibility criteria; or (2) alternatively bracket creep occurs where eligibility criteria is expanded and so those accessing AS/E no longer fit medical criteria (e.g., elderly and “tired of life”), or it is expanded to “greyer” areas of “medical criteria” such as psychological distress, which has come to be classified as illness and can be treated as such, but is also clearly underpinned by more complex social determinants of health that can be treated without medical intervention and is often reversible.^39^ Bracket creep thus presents pragmatic problems for doctors in terms of both their personal ethics, as well as in terms of knowing where their professional boundaries lie regarding who can access AS/E in legalized jurisdictions where terminology is more ambiguous. This latter point presents a legal problem because what counts as medical criteria concerning “suffering” (and whether this encompasses psychological suffering or tiredness of life) means that doctors remain exposed to possible criminal law repercussions if they overstep the boundary of what the law deems acceptable eligibility criteria.

More than this, though, medicalization individualizes social problems and risks removing the burden on society to tackle fundamental social inequalities that might drive some people into seeking out AS/E, and it also risks the prospect that AS/E becomes subject to healthcare economics. I examine these points more fully in the next sections.

## Medicalization in Canada and the Netherlands — restrictive and expanding criteria

A particular challenge facing Canada’s legislation of assisted dying via its MAiD model is the ambiguity of its criteria that has permitted its expansion. Introduced initially in 2016, MAiD was restricted to persons with “reasonable foreseeability of natural death” (RFND) and was intended to provide AS/E to persons with serious and incurable illness, disease or disability, which was in an advanced and irreversible state of decline, and which caused intolerable suffering that could not be alleviated under conditions the person considered acceptable.^40^ However, in 2019 people with disabilities, specifically Nicole Gladu, who lived with an incurable degenerative disease called post‐poliomyelitis syndrome, and Jean Truchon, who lived with cerebral palsy, challenged the RFND restrictions, arguing them to be discriminatory. Quebec Justice Christine Baudouin agreed and in her Superior Court’s decision in *Truchon*, deemed the RFND criterion unconstitutional.^41^ Following *Truchon*, Canada’s “Track 2” for Non-RFND (NRFND) expanded MAiD to Canadians with disabilities who are not dying. Data from Ontario’s coroner’s reports on MAiD reveals that those with NRFND typically present with complex conditions such as chronic pain and neurological conditions.^42^ The development and expansion to Track 2 has attracted notable criticism. Lemmens has described the legal development of MAiD as the normalization of the view that there is an “unrestricted constitutional right to MAiD.”^43^ Despite Canadian law requiring a “grievous and irremediable medical condition” as a precondition for MAiD, the country’s rights-based approach has largely meant that MAiD is effectively on-demand and has contributed to a culture of normalization MAiD as part of healthcare. Lemmens notes how the Canadian Association of MAiD Assessors and Providers proactively recommends MAiD to patients who do not even request it but who might qualify. This has led to controversial cases including a Nova Scotian woman with an underlying autoimmune condition who was advised of MAiD when preparing for mastectomy surgery.^44^

The normalization of MAID is particularly troubling for Track 2 recipients. Grant, for example, has argued that it “falls afoul of the legal standard.”^45^ Track 2 medicalizes disability, treating it as a condition to be fixed. When state funded death is available to people with disabilities alongside a state failure to provide adequate social support and housing to people with disabilities, AS/E is not an individual choice. As such, Track 2 MAiD conflicts with The Convention on the Rights of Persons with Disabilities (CRPD) and sections 7 and 15 of the Charter. Equality law could therefore be invoked to argue against some forms of legalization of AS/E, Grant argues.^46^ Indeed, the UN Special Rapporteurs have also raised concerns that assisted dying may institutionalise “ableism” and could violate Article 10 of the CRPD because it might undermine the lives of disabled people.^47^

Earlier I noted the issue of medicalization in relation to the expansion of eligibility criteria, known as “bracket creep.” One may critique medical models for bringing otherwise “normal” life feelings and experiences (like disability) into the domain of medicine. Likewise, concerns over further expansion of the Canadian criteria to include those with mental illness may complicate medical practitioners’ ethics and practice of AS/E even further.^48^ For example, permitting AS/E for mental illness opens the prospect that AS/E will become available for persons with anorexia, for instance, which has been seen in Oregon.^49^

The medicalization of death is not only important in terms of the management of AS/E, i.e., is who is then tasked with performing it, but it is also shapes how individuals come to give their own lives and deaths meaning and value. One could argue that mental suffering and ill health is already an elusive issue that is frequently medicalized. For instance, Davis argues that the medicalization of mental health is not only fueled by pharmaceuticalization, underscored by modern capitalism, but that it furthermore individualizes mental health concerns (i.e., the notion of the “broken brain”) and thus focuses us away from the social inequalities driving poor mental health.^50^ Even if societal pressures might trigger psychological states of suffering, like stress at work caused by precarious working conditions, for instance, most of the global northern world considers individuals suffering with mental health concerns to be medical patients who require treatment rather than focusing on finding solutions in the social and structural conditions driving poor mental health. Ideally, we would address both the individual and the societal causes of mental illness.

Expanding AS/E to persons with mental illness has also led scholars to question whether there is a contradiction between MAiD and the public health goal of suicide prevention. If the aim of public health is to improve people’s lives and to also alleviate structural inequalities, does a healthcare system that also facilitates an assisted death not present incompatible messages?^51^ One might argue that such a contradiction may be diluted (though perhaps not necessarily resolved) in a demedicalized AS/E model that sits outside of healthcare. The expansion of eligibility criteria into areas that are not typically of medical jurisdiction via processes of medicalization presents ethical as well as potential legal problems for medics. Ethically, the bracket creep presents a problem for medical professionals who agree with performing AS/E as a means to end some forms of suffering for patients whose death is reasonably foreseeable, but who may be less inclined to do so for patients whose cases are more complex. Evidence suggests, for example, that doctors may be more inclined to provide AS/E for persons who they deem to be medically ill but are less inclined to perform it on persons with a mental illness.^52^ Some doctors even report these latter types of requests as morally distressing.^53^ If AS/E sits in the statutory space of medicine, then doctors become duty-bound to provide it to eligible patients who want it, even if they have reservations.^54^ Legally, expanding AS/E to areas such as disability with NRFND, may also leave doctors in a grey zone if Track 2 is deemed unconstitutional, as Grant argues.^55^ Managing AS/E outside of healthcare to permit non-medical suffering may therefore not only be deemed by some to be more equitable but may also circumvent some of the concerns that doctors have regarding its expansion within medical models of provision.

## Bracket creep and expanding criteria – making a grey zone even greyer

If medicalization in the context of assisted dying is taken to refer to the treatment of otherwise non-medical issues as medical problems through AS/E, the Netherlands is a strong example of such a medicalizing trajectory. Kimsma,^56^ one of the leading commentators and advocates of the Dutch euthanasia system has suggested that there has been “undeniable expansion” of AS/E into the domain of medicine including to patients with Alzheimer’s, chronic psychiatric disorder and a “completed life.” The 2020 Dutch bill aimed to extend euthanasia to anyone over 75 who requests it. Even if very few cases in practice emerge from those who are “tired of life” and thus present no underlying medical condition, arguably the expansion of AS/E to this criterion normalizes AS/E in a realm of medicine as a “treatment” by bringing otherwise-considered “normal” life “experiences” and sentiments (such as general feelings of a tiredness of life) into the realm of medicine.

Such a point was also identified in the 1994 Netherlands Supreme Court case of *Office of Public Prosecutions v Chabot.* ^57^ In this case, the psychiatrist Chabot assisted a woman, Mrs. B, to kill herself because she was suffering from the grief of the death of her two sons. Chabot was subject to the charge of performing euthanasia. Until 2002, the Netherlands had legalized euthanasia but had not decriminalized practices that did not meet the legal demands of the “rules of careful practice.” In decisions preceding Chabot, the Dutch Supreme Court had recognized a defense of necessity, only under narrowly defined circumstances, to this charge. Whereas historically the courts had dealt with this defense of necessity in relation to somatic illnesses, and in a terminal phase, Mrs. B’s case raised a different issue which was whether this necessity defense could be used for cases where a subject presents with psychiatric or psychological illnesses. The court thus considered whether Mrs. B had suffered any somatic illness or was in a terminal phase that would justify her assisted death. The defendant, Chabot, argued that Mrs. B entered into a doctor-patient relationship as she sought help for severe mental difficulties. While she was not psychotic, she was deeply distressed. Chabot argued the defence of necessity: that it was necessary for him to help Mrs. B end her life. While Chabot was deemed not guilty, the case was appealed on the grounds that the patient did not suffer somatically and was not terminally ill, and that a psychiatric patient cannot act voluntarily (in Mrs. B’s case, there was no second psychiatric opinion). The Supreme Court decided that the Court of Appeal and the earlier District Court should have rejected Chabot’s defence.^58^ From a legal perspective, crimes are determined through the precedent set in early cases. The fact that Chabot’s case incorporated something new — in Mrs. B’s case, “non-somatic” suffering — was considered problematic.

As Griffiths^59^ notes, the Chabot case created an opening for other “types of subjects” who were not terminally ill or showing somatic symptoms to be incorporated into the medical system were they to feel troubled — such as elderly persons who were “tired of life.” The medicalization of decisions over death cast the net of the doctor-patient relationship even wider, opening up the prospect of medicalizing otherwise “normal” mental states: feelings of distress from social and environmental precursors, and feelings of tiredness of life. Indeed, Chabot himself even noted as much in his later reflections, where he has openly expressed concern about the rise in euthanasia for patients with chronic psychiatric diseases and dementia.^60^

Because suffering is a subjective concept, it leaves physician discretion and thus judicial discretion open. Increasingly doctors are concerned about how the creep of AS/E criteria might weigh even more heavily on doctors’ consciences. Keown^61^ has noted the expansion of medicalization at the end of life towards decisions over the termination of infants suffering from severe illness or disability in the Netherlands, for example.

Since *Chabot*, the Dutch courts have seen other cases of AS/E come to the fore that have tested the boundaries of medicalization. Such was the case in the Brongersma case which, in 1998, involved a former lawyer and senator who was an AS/E advocate and wrote a living will, requesting AS/E on the grounds of suffering.^62^ Eighty-six year old Brongersma had previously attempted suicide and had updated two living wills requesting AS/E. Brongersma contacted his doctor, who in turn contacted an independent psychiatrist to assess his case. The psychiatrist did not find Brongersma to suffer from psychiatric illness that could constitute medical grounds for AS/E. However, the first doctor sought a second opinion from a different physician, who agreed that Brongersma’s appeal to die was voluntary and permissible. The case pushed the boundaries of what the law considered acceptable degrees of medicalization. The district court concluded in Brongersma’s case that suffering could be derived from non-medical causes and thus the doctor (GP Philip Sutorius), who assisted, was acquitted. However, the prosecution appealed on two counts — one because of concern that the case would incite an expectation to self-determination and two because of doubt over the “unbearableness” of his suffering.^63^ Subsequently, the Dutch Court of Appeals in Amsterdam found Sutorius guilty of assisted suicide because the case did not meet the medicalized suffering criteria, though no punishment was imposed upon him. Sutorius appealed to the Supreme Court against his conviction, which was dismissed.^64^ The issue here is that doctors do not necessarily know their legal foothold and therefore are exposed unfairly to the criminal law.

## Is “demedicalization” an option? The barrier of “pharmaceuticalization”

If medical models are unsatisfactory because they are either too narrow in their eligibility criteria (restricted to medical criteria), or are too broad such that they move outside and thus broaden the remit of what is deemed a “medical” issue (medicalization), or even expose doctors to grey areas of criminal law, an alternative is surely to “demedicalize” AS/E. Demedicalization has been defined as the process of “stripping away medicine as a dominant frame of reference.” Ost provides the useful example of childbirth to illustrate demedicalization, noting the turn away from hospitalized forms of childbirth to a reclamation of non-medical techniques^65^ (such as hypnobirthing). Palliative care and the natural death movement are also examples of demedicalization.^66^

In the field of AS/E, Ost suggests there is evidence of a trend towards demedicalization. Suicide tourism to places like Switzerland where assisted suicide takes place outside of hospital settings highlights more familial involvement in the process of helping family members access an assisted death and offers an alternative frame of reference to appeals for clinical deaths.^67^ Indeed, Switzerland presents an interesting counterexample to the medicalized AS/E trend. Despite Swiss law permitting assisted suicide if it is “inspired by altruistic motives,” assisted suicide in Switzerland is largely regulated in ad hoc form by a group of “right to die” organizations.^68^ There is no statutory legal framework that sets out positive rights or obligations of the state to provide assisted suicide (not euthanasia). Instead, Switzerland’s framework is “decriminalized” — it is permissive but deregulated. Andorno^69^ has pointed out that the Swiss laws on assisted suicide were not the result of a liberalizing government policy so much as the result of opportunism on the part of nonprofit organizations (NPOs) who saw a legal gap in the Swiss Penal Code which enabled them to act as service providers. Four primary right to die NPOs manage and regulate assisted suicide in Switzerland, although several smaller, newer organisations have emerged. Dignitas is the most well-known by the international public because its services are available to non-Swiss residents.^70^ Exit data indicates record-high numbers of organization members, which the organisation attributes to an ageing society and high numbers of people with disability and illness.^71^ Generally, NPOs derive their income from membership, bequests and donations.

Swiss law also clearly differentiates between assisted suicide and euthanasia, permitting the former, but continuing to criminalize the latter, which remains punishable under Article 111 (murder) and Article 114 (mercy killing on request) or Article 113 (manslaughter) of the Swiss Penal Code.^72^ Although Swiss law permits AS (not E), in its earliest 2004 guidelines (in the Care of Patients in the End of Life) the Swiss Academy of Medical Science (SAMS) had stipulated that assisted suicide was not part of a physician’s duty.^73^ This has sometimes been interpreted to mean that physicians should not assist with death, but this was not strictly the case. Instead, Swiss physicians were treated like laypersons under the Swiss law, and they had the same discretion as any citizen to assist with death; it simply removed the obligation to do so from their professional role.^74^ This has led to the Swiss model being sometimes referred to as a “demedicalized” model.^75^ Although this remains largely accurate, as Halfmann argues, the process of medicalization and demedicalization is complex: often both concepts are at play at once and these are best understood as processes and thereby one could argue degrees of medical influence, rather than discrete categories into which practices do or do not fall.^76^ Returning to the example of childbirth, for instance, as Conrad notes, although there has been a move away from medicalization with some mothers opting for more “natural” births, most instances of childbirth in the global north still take place in hospital settings.^77^ Likewise, end of life care in hospices might be less institutionalized than deaths in hospital settings, but deaths are rarely doctor-free. Demedicalization might describe perhaps less medical involvement, but this is a matter of degree rather than absence. One area largely ignored, but arguably a great challenge that keeps Swiss AS/E bound with medicine, is its pharmaceuticalization.

Abraham defines pharmaceuticalization as “the process by which social, behavioural or bodily conditions are treated or deemed to be in need of treatment, with medical drugs by doctors or patients.”^78^ Arguably, pharmaceuticalization is a largely under-researched area of the AS/E regulatory debate but it is central to the continued medicalization of AS in Switzerland. Besides the dearth of research into the efficacy and safety of AS/E drugs or other lethal methods for performing assisted suicide,^79^ there is pragmatic issue of how pharmaceuticalization binds medicine to Swiss AS. Although Switzerland does not stipulate strict eligibility criteria for persons receiving AS and there are no restrictions relating to the grounds of suffering, the laws surrounding dispensation of therapeutic products regulates AS access. Pharmaceutical laws govern who can prescribe medication (only doctors); therefore, although AS in Switzerland does not take place in healthcare settings, the process remains embedded in a medical model.

Examining the Swiss criminal law also gives insight into how the Swiss treat AS for “existential suffering.” Different cantons have drawn up different guidelines, though in more recent years the Swiss Federal Court has also served to clarify this grey area through judgments on two well known cases. In the first judgment concerning Dr. Erika Preisig, the president of smaller NPO, Lifecircle, Preisig was accused of murder and for violating the Therapeutics Products Act for assisting a mentally ill patient to die without obtaining the opinion of a specialist.^80^ The cantonal court dismissed the homicide charge which was later appealed by the prosecutor and referred to the Federal Court which, in 2023, ruled in favour of Preisig and clarified the laws on assisted suicide further. It stipulated that so long as mentally ill persons’ decisions are well considered, it was permissible to assist a mentally ill person die and that a psychiatric report was not mandatory. In the second judgment, former vice president of Exit ADMD Pierre Beck and a Swiss doctor, were also charged with helping a “healthy” person die. The case involved an 86-year-old woman who wanted to die along with her husband. Geneva’s cantonal criminal appeals court had judged Beck’s actions to be a serious offence and he was fined, but this was later overruled in 2021 by Switzerland’s Federal Supreme Court which demanded that the doctor was retried under the narcotics administration laws at the cantonal court, which then ruled that the law on narcotics does not punish the prescribing of pentobarbital to persons in good health.^81^

As noted earlier, although the Swiss laws do not restrict assistance on the basis of eligibility criteria for assisted suicide, doctors, who may act as citizens, are bound by laws governing prescribing as well as by their medical association’s guidelines, and although the medical guidelines are not legally binding, doctors who violate them can be struck off the medical register, losing their licenses.^82^ Swiss regulation also presents a unique model in that AS is decriminalized but is not legalized. This means that there is no positive “right” to AS in Switzerland; it also means that the criminal law continues to regulate AS more heavily than other jurisdictions where it is regulated within healthcare. Although AS is normalised as a practice in Swiss NPOs, the continued investigative process involving the police and Institute of Legal Medicine representatives suggests that assisted dying regulation in Switzerland continues to rely quite heavily on the criminal law.^83^ When tested on its positive obligation to provide assisted suicide, the case of *Haas v. Switzerland^84^* revealed that Swiss law provides no positive right to an assisted suicide. In this case, a 57-year-old man suffering from bipolar disorder had appealed to the European Court of Human Rights that his Article 8 right had been infringed upon because he could not achieve a prescription for pentobarbital to access an assisted death. He claimed that he had contacted psychiatrists but could not find someone willing to prescribe for him and that in exceptional circumstances like his, the state should be able to provide the substance. The Swiss government suggested that because others in similar circumstances had historically accessed such a substance, he should have been able to find someone willing and, if he had done so, his assistors would not have been prosecuted — as prior cases had also revealed. Yet, the Swiss government also argued that it was under no obligation to provide this access and could not directly license this medical prescription.

## Between medicalization and demedicalization

The extent of Swiss medicalization of assisted suicide — i.e. the degree to which a doctor has historically interpreted “medical” need as a rationale for drug prescription — is grey.^85^ Medical codes and intersecting laws also regulate the behaviour of doctors who interpret these codes and laws. Competing discourses make for complex decisions for doctors who are sometimes torn between the normalization of assisted suicide and wider concerns shaped by contrary discourses, sometimes religious, or punitive, that different subjects internalize and act upon. For example, some doctors may internalize demedicalizing discourses and therefore they may choose not to prescribe for cases of assisted suicide, while other doctors may limit what they regarded as a medical issue to illness or disability, and others, still, might extend medicalization to the full limits of the law and included those people who are “tired of life.” This suggests there is room for doctors to make their own professional decisions about when and whom to permit AS for in the Swiss model, but it also means medics’ judgments may be parochial and not necessarily equitable.

Some scholars suggest that the Swiss demedicalized model is preferable to the Dutch because it sidesteps wider concerns around medicalization, raised above.^86^ Ziegler argues that the Swiss model that enables “right to die” organisations to partake in assisted death provides more transparency in the process of assisted death.^87^ Attempts to demedicalize AS/E, however, also risk sequestering it further into the private sphere of the family and away from state control and thus also risk the increased view that AS/E is a personal trouble to be dealt with behind closed doors rather than as a public health issue that also requires focus on structural vulnerabilities, a point I turn to in the next section.

In sum, demedicalization poses two primary risks: first, the sequestering of AS/E from healthcare into the private/NPO sphere risks a lack of transparency and oversight. The partially demedicalized model that exists in Switzerland also presents the need for more continued involvement from the criminal law. Second, demedicalization risks deregulation and a lack of legal certainty: without central oversight and legal bright lines, doctors operate in legal grey areas. Swiss AS is governed by “social mores”^88^ and because these tend to shift it is not always possible to know where the bright line is drawn. A final factor raised in this section concerned pharmaceuticalization. Any jurisdiction considering a model of AS/E outside of healthcare, or considering moving towards demedicalization, would have to consider the hurdle of prescribing, and the prospect that pharmacists could supply AS/E drugs to licensed, non-medical personnel.

## Does medicalization depoliticize public issues?

Where the above sections have considered the pragmatic issues of doctor objection, and the challenges with defining suffering as a medical issue as well as the complex relationship between demedicalization and pharmaceuticalization, this next section focuses on the concern that medicalization “depoliticizes” public issues. Depoliticization refers to the emphasis of looking “for causes and solutions to complex social problems in the individual rather than in the social system.”^89^

Borrowing from C. Wright Mills’ classic “The Sociological Imagination,”^90^ it is important to reframe AS/E requests, often deemed to be “personal troubles,” as “public issues.” Mills argued that personal troubles are private problems that individuals experience whilst public issues are societal issues that affect many people at once. Arguably there is a tendency to focus on AS/E as a personal trouble or individual problem. Medical models of AS/E regulation reinforce this view: they emphasize the individual requesting AS/E as having a problem to be cured or treated, rather than focusing on the broader structural reasons that sit behind the request. In turn, medical models of AS/E regulation can detract from a political focus on the wider structural issues that underpin the so-called “medical” problem. A sociological approach to AS/E regulation would not only consider how wider social determinants of health and illness are contributing factors that lead people to assisted suicide but would instead place the question of structural vulnerability and social inequality at the center of the debate.

Some research has explored the relationship between AS/E recipients and socio-structural conditions. However, the research in this area is contradictory. On the one hand, some scholars like Downar et al. argue that socio-economic factors are not drivers for AS/E.^91^ Other scholars also point to data that suggests most people seeking AS/E are older, middle class, white males, primarily diagnosed with cancer.^92^ Swiss,^93^ American^94^ and Canadian^95^ data also links higher socioeconomic status (SES) groups to AD applications. This is a more traditional view of AS/E, which is to say that it is a “middle class” and also “white” global northern problem, and not a problem affecting society’s marginalized.^96^ Despite the evidence that points away from low SES linkages to AS/E, contrary data exist. In a response to Downar et al.’s research, signed by over 170 scholars and practitioners, the authors describe the “evidence” as misleading and underpinned by cherry-picking data to confirm a point of view.^97^ Longitudinal data from Oregon, for instance, reveals that categories of those accessing AS/E shift over time with cancer diagnosis a declining category, from 80% in the first 5 years to 64% in 2022, with other groups increasingly receiving AS/E.^.^^97^ Longitudinal analysis also reveals that socio-economic factors increasingly influence AS/E decisions, including feelings of being a burden.^98^ Other Canadian data reveal that medics are seeing poverty as a “driver” for MAiD. Tran et al.’s research, for instance, reveals low SES groups comprise a disproportionate number of Canadian MAID applicants, although they receive a similar proportion of assistance to other SES groups.^99^

Qualitative data from Belgians seeking euthanasia for psychological suffering revealed socio-economic problems including lack of finances, feelings of being a burden and social isolation.^100^ Such points are corroborated in data from Ontario’s coroner’s reports where MAiD recipients were in higher levels of poverty than the population (28.4% of Track 2 MAiD recipients (NRFND), 21.5% of Track 1 recipients (RFND) compared to 20% of the population). 48.3% of Track 2 and 34.3% of Track 1 recipients were in the worst housing instability quintile, and 56.9% of Track 2 and 41.8% of Track 1 recipients were from the most vulnerable quintiles related to age and labor force participation.^101^ Furthermore, 38.8% of recipients in Track 2 noted the perceived burden on family, friends, or caregivers as a description of intolerable suffering, and isolation or loneliness were also considered components of intolerable suffering, particularly for 39.7% persons in Track 2.^102^ A small qualitative study that conducted interviews with 20 MAiD providers also revealed that although the providers had not personally experienced many cases where patients requesting AS/E had unmet needs, when they did these usually related to loneliness and poverty, and could lead to ethical dilemmas for providers who recognize that suffering is partially caused by social and structural failures.^103^

Gathering appropriate and relevant statistical data is therefore essential to ensure accountability of any AS/E regime. However, scholars have also noted that monitoring is often ineffective. For example, Oregon has missing data,^104^ and Canadian data is not collected objectively; the data gathered by the latter’s oversight body is criticized as “box ticking” rather than capturing the detail and experience of those who are socially vulnerable which emerges through qualitative research.^105^ Scholars have thus identified that any data collection methods must be readily available, able to detect problems and must be able to easily identify any concerns around structural vulnerability and AS/E.^106^

As some authors point out, relying simply on demographic data to make correlations between socio-structural vulnerabilities and AS/E misses finer grained detail as well as broader social and contextual factors concerning who ends up seeking AS/E.^106^ People in higher SES groups with higher levels of education tend to navigate the healthcare system more easily and are more able to access support and medical resources, including palliative care, than lower SES and less educated persons. This may be particularly important in jurisdictions that do not restrict eligibility to a reasonably foreseeable natural death. Furthermore, relying simply on statistical correlations misses broader sociological insights into the stigmatization of certain groups, including persons experiencing severe and debilitating disabilities, or those who are elderly and infirm, groups who are among the most likely to seek AS/E.^107^

For example, research has explored the impact of the medicalization of disability and how it can reinforce ableism when disabled people are seen as having something wrong with them that requires fixing through medical intervention.^108^ Canada, for instance, has seen increasing reports of disabled people being offered unsolicited MAiD.^109^ The normalization of AS/E medicalization for persons with disabilities may risk institutionalizing “unconscious discrimination,”^110^ both in doctors who may come to see persons with disabilities as “natural” recipients of AS/E, and in the social care system which may see AS/E as a viable alternative to providing greater care and support in living. This is of course not to deny that persons with disabilities may have the capacity to consent to AS/E but rather to acknowledge from a sociological perspective that structural norms persist that might shape personal choice.

Drawing on the medicalization literature one could also argue that even though most current requesters are not typically marginalized — i.e., that AS/E in structurally vulnerable populations is rare — the continued medicalization of AS/E as a personal trouble risks pushing more people who are structurally vulnerable toward requesting AS/E as a solution to their social marginalization. One must therefore consider the socio-economic context in which the medicalization of AS/E emerges and in which AS/E decisions are made: to what extent does the decline of the welfare state and the subsequent privatization of healthcare, and the demands in old age to live for longer but to continue living alone without burdening others, remain entrenched in our day-to-day discourses, shaping people’s “personal” choices?

Decisions to medicalize AS/E are not neutral; instead, they are heavily laden social decisions, weighed up within larger discussions of the economy, social norms, and population management. As Keown adds, many persons deemed to fulfil the requirements for assisted death are framed as already “better off dead.”^111^ It was Baroness Mary Warnock, a member of the English House of Lords and a strong advocate of euthanasia, who famously noted that the elderly and ill who burden their families ought to “creep off and get out of the way.” In a 2004 *London Times* interview she noted: “in other contexts, sacrificing oneself for one’s family would be considered good. I don’t see what is so horrible about the motive of not wanting to be an increasing nuisance.”^112^ Some academics like Kissel^113^ support this position, whilst others like Hardwig^114^ question the ethics of such a position that may compel some subjects to seek assisted death in order to relieve loved ones of the burden they feel they impose onto them.

Supporting an individual’s right to choose AS/E in medical contexts where an assisted death is possibly framed as a more humane or dignified death must therefore also be balanced with an understanding of how requests for AS/E take place within social conditions that normatively frame some subjects as abject and more suitable for death. In many requests for AS/E, people speak of the “indignity” they feel when requiring assistance with personal care such as toileting (e.g*., R (Nicklinson) v Ministry of Justice).* ^115^ Certainly, in global northern societies where individual choice and independence are core values, it is clear why persons might regard more dependent forms of existence as undesirable. These are, however, contextual values rather than moral facts. While many people want a “choice” to request AS/E, this choice is shaped by wider social norms and pressures that people will arguably internalize. Subjectivist accounts of autonomy thus fail to acknowledge, or decide to override, the reality that social inequalities are contributing factors to appeals.^116^ This is not to suggest that choices cannot be made, or that rights to die should not be given, but instead it is to draw attention to how autonomy and “choice” may conform to wider socially normative notions of the good life and, thus, too, the good death.

One particular challenge of medical models regulating AS/E is how they can ensure that structural vulnerabilities are feasibly assessed and integrated fully into the medical curricula and into practice. Evidence suggests this has proven challenging.^117^ Expecting all medics to be sociologists and ethicists, on top of already overburdened workloads may be too much to ask.

Socially normative notions of the good life are also not divorced from healthcare economics and personal financial decisions. The question as to whether AS/E would be more cost-effective for governments and the taxpayer rather than ongoing care such as specialist palliative care is one that ought to remain public facing. Once again, however, it is important to openly acknowledge the competing evidence that is presented in political debates, and that language is never neutral.^118^ For example, Australian data draw from research evidence that claims AS/E provision does not impact palliative care spending,^119^ while data from Benelux countries indicate a stalling of palliative care spending.^120^ The former evidence was cited in the recent UK Health and Social Care Inquiry into Assisted Dying, yet the latter data were omitted from discussion.^121^ A transparent discussion of healthcare economics is essential in this debate as it is often one of the most cited reasons for vulnerable persons’ concerns over AS/E legalisation. For example, in the jurisdiction I am writing from — England — cost-savings is an important question because demographic trends highlight longer lifespans but with more comorbidities including disability, leading to rising demands for social care provision: evidence reveals increasing numbers of over 65s requiring care, living with life limiting conditions and disabilities. The Care Policy and Evaluation Centre (CPEC) has projected that these demographic pressures will increase adult social care demand and expenditure by 43% from 2018 to 2038.^122^

Evidence from Switzerland also reveals that tensions exist in nonprofit organisations (NPOs) between a social value orientation, and the requirement that NPOs can self-fund. NPOs must function as entrepreneurial businesses to survive in a marketplace offering similar services. Although volunteers work for these organisations, NPOs also have paid employees with salaries and thus are business entities, with average costs to use Dignitas’s services culminating to circa £10,000.^123^ Despite being cleared of charges, Dignitas founder, Ludwig Minelli, was accused in 2018 of exploiting patients and profiteering after public prosecutors presented evidence that he had sought out four different physicians to find one who would provide a lethal prescription to an elderly French woman, who had entrusted, upon her death, a 100,000 franc donation to Dignitas. Switzerland has a history of a limited state and larger degrees of civic involvement, but this is not divorced from advanced liberal governance regimes, and NPOs operate as decentralized solutions to provide welfare needs which the government does not itself fund. Some academics have attempted to estimate the proceeds from assisted suicide as around £53 million annually.^124^

Similarly, adequate access to support for terminally ill persons would need consideration. Evidence suggests that families including someone with a terminal illness face significant financial pressures and the current welfare provision is inadequate — in the UK, access to benefits is only possible if someone has less than 6 months to live — which may also present assisted dying as a way out of financial trouble and a desirable option when there appears to be no alternative.^125^ Also in the UK, a cancer diagnosis correlates with an income of £570 per month less on average,^126^ and in the USA a cancer diagnosis increases the risk of bankruptcy by 250%.^127^ Although some research evidence claims that AS/E does not decrease government expenditures on end of life care — an argument sometimes put forward by “slippery slope” proponents to argue against AS/E^128^ — other evidence suggests that it is important to consider how AS/E could be seen as a way out of an individual’s financial hardship.^129^

Several recommendations can be drawn from this section: (a) that appropriate data are collected in countries where AS/E is performed that specifically monitors not only AS/E rates and demographic variables but that also captures structural vulnerabilities to ensure these do not become mitigating factors in requests for AS/E. (b) Countries also need to consider how disability rights can be protected under equality laws and this may mean re-examining whether current or future AS/E provision may be unconstitutional. (c) If AS/E remains regulated in healthcare settings it would be important to examine how doctors may better integrate structural vulnerabilities in their assessment of AS/E requests; (d) in the same way that palliative care has been sequestered from hospitals to hospices, outside the mainstream function of medicine to “cure,” so too might AS/E provision be sequestered into a different area of healthcare (or outside of it) that is distinct from palliative care and that is given sufficient time, resource, and remuneration.

## Conclusions

This paper has argued that despite a medical model being the dominant frame of reference for AS/E provision and regulation, a variation of a demedicalized model is possibly a more pragmatic alternative due to the various issues raised in this paper concerning medical models including: possible objections to, and thus decreased efficacy in medical service delivery; the challenges of managing bracket creep within medical criteria that are either too restrictive or unsuitable; to the complications that come when we mix up end of life care with healthcare economics which might be more ethically sequestered to keep these areas more discrete.

The Swiss model is sometimes referred to as an alternative demedicalized model, but this paper has argued that it is far from demedicalized, largely because it remains bound to medicalization through the laws concerning therapeutic products, part of a larger issue concerning “pharmaceuticalization.” One area to consider further is whether laws could be amended across different jurisdictions to facilitate demedicalized models, while also accounting for some of the ethical and legal challenges of demedicalization. The emergence of the Sarco pod, a 3D-printed suicide chamber in Switzerland, is one example where people have been enabled to commit suicide without medical intervention through the use of technology.^130^ This alleged solution to medicalization, however, avoids dealing with the socio-structural inequalities entirely: it only serves to reify structural vulnerabilities and social marginalization rather than bring them to the forefront of regulatory discussions as this paper has suggested. Any demedicalized model would have to consider whether non-pharmaceutical alternatives for AS/E are viable, or whether there is a model for AS/E regulation that could sit outside of healthcare but that could overcome the challenges the Swiss face with regards to pharmaceutical regulation and restrictions, and indeed whether these solutions may be deemed more or less ethical than the current medical models in existence.

The paper has also argued that to consider demedicalization as the opposite of medicalization also risks turning concepts into categories rather than recognising the continued intersection and overlapping nature of medical practice in society at large. To divorce these areas would be practically impossible. However, there are ways forward to consider more pragmatic and ethical ways of managing and regulating AS/E globally. Empirical evidence from states in Australia suggest that medics are overburdened and are expected to perform AS/E to patients on top of normal workloads, which may limit uptake of providers. Evidence elsewhere reveals that medics feel conflicted, particularly in cases dealing with greyer areas of psychological suffering, that doctors who object to AS/E may experience bullying, and that these values held by medics impact directly on patients. When persons who perceive they have a positive legal “right” to die are unable to access AS/E in a timely and uncomplicated manner this can undermine doctor-patient relationships in already fragile and overstretched healthcare services.

In sum, AS/E regulation is a complex debate, underpinned by various and sometimes contradictory sets of evidence, competing political agendas, and diverse sets of beliefs, that requires a nuanced and politically balanced solution. As this paper has revealed, it is essential to consider empirical data sets as evidence of successful and unsuccessful models of AS/E regulation. More than this, it is important to consider how empirical data are collected in a wider social context — that is, how evidence is subject to framing and interpretation bias. Although this paper does not draw a firm conclusion about whether AS/E should sit inside or outside of medical jurisdiction, it draws attention to the challenges of the dominant medical model, particularly as it applies to the creep of medicalization into traditionally non-medical areas of life. Furthermore, framing the debate in the context of medicalization and pharmaceuticalization reveals the challenges of sequestering AS/E away from medicine. There appears to be a comfort in medical models that position them as safe spaces for AS/E, which this paper challenges in terms of the pragmatic realities of such a belief.

A pragmatic solution may be to consider how AS/E can be better sequestered from healthcare, and its operations performed by people who come to specialize in it as a subfield. A pragmatic solution would also need to protect against the possibility that in both medicalized and demedicalized models, social problems do not continue to simply be treated as personal troubles but that instead any regulatory mechanism keeps good oversight of social demographic variables, ensures that people choose the service willingly, and that greater social care provision is centered in government policies where AS/E becomes an option. As Busfield writes, “defining a condition as an illness and adopting a medical approach can have major social consequences and close off alternatives,”^131^ but equally, medical models might have some advantages as this paper has discussed, and these must not be lost if jurisdictions move further towards demedicalization.
